# Cell Membrane Biomimetic Nanoparticles with Potential in Treatment of Alzheimer’s Disease

**DOI:** 10.3390/molecules28052336

**Published:** 2023-03-03

**Authors:** Xinyu Zhong, Yue Na, Shun Yin, Chang Yan, Jinlian Gu, Ning Zhang, Fang Geng

**Affiliations:** 1 School of Chemistry & Chemical Engineering, College of Chemistry & Chemical Engineering, Harbin Normal University, Harbin 150025, China; 2 College of Jiamusi, Heilongjiang University of Chinese Medicine, Harbin 150004, China; 3 Wuxi Traditional Chinese Medicine Hospital, Wuxi 214071, China; 4 Wuxi School of Medicine, Jiangnan University, Wuxi 214122, China

**Keywords:** Alzheimer’s disease, blood–brain barrier, cell membrane biomimetic nanoparticles, targeting peptides

## Abstract

Alzheimer’s disease (AD) is to blame for about 60% of dementia cases worldwide. The blood–brain barrier (BBB) prevents many medications for AD from having clinical therapeutic effects that can be used to treat the affected area. Many researchers have turned their attention to cell membrane biomimetic nanoparticles (NPs) to solve this situation. Among them, NPs can extend the half-life of drugs in the body as the “core” of the wrapped drug, and the cell membrane acts as the “shell” of the wrapped NPs to functionalize the NPs, which can further improve the delivery efficiency of nano-drug delivery systems. Researchers are learning that cell membrane biomimetic NPs can circumvent the BBB’s restriction, prevent harm to the body’s immune system, extend the period that NPs spend in circulation, and have good biocompatibility and cytotoxicity, which increases efficacy of drug release. This review summarized the detailed production process and features of core NPs and further introduced the extraction methods of cell membrane and fusion methods of cell membrane biomimetic NPs. In addition, the targeting peptides for modifying biomimetic NPs to target the BBB to demonstrate the broad prospects of cell membrane biomimetic NPs drug delivery systems were summarized.

## 1. Introduction

The most prevalent type of dementia in the world is Alzheimer’s disease (AD), which is a progressive and irreversible neurodegenerative disease [1]. The possible predisposing factor of dementia in elder citizens is neuronal loss and cognitive impairment brought on by buildup of aggregation of amyloid-β (Aβ) and intracellular neurofibrillary tangles with aging [2]. According to World Health Organization projections, more than 55 million people worldwide already suffer from dementia, and that number is projected to increase to 78 million in 2030 and even more than 139 million in 2050 [3]. The researchers also found that the median survival time of AD diagnosis age was 3.2 to 6.6 years [4], and the probability of heritability was about 70%. As the number of AD patients increases, the situation facing patients and their families is dire [5]. A number of drugs for AD have also advanced to the clinical research stage, costing billions of dollars [6]. However, 98% of drugs currently available on the market are blocked by the blood–brain barrier (BBB) in treating AD [7], which prevents pathogens and drugs from entering the brain from the circulatory system [8]. Drug delivery that targets the central nervous system for diagnosis and treatment of neurodegenerative diseases such as AD is restricted.

**Figure 1:**
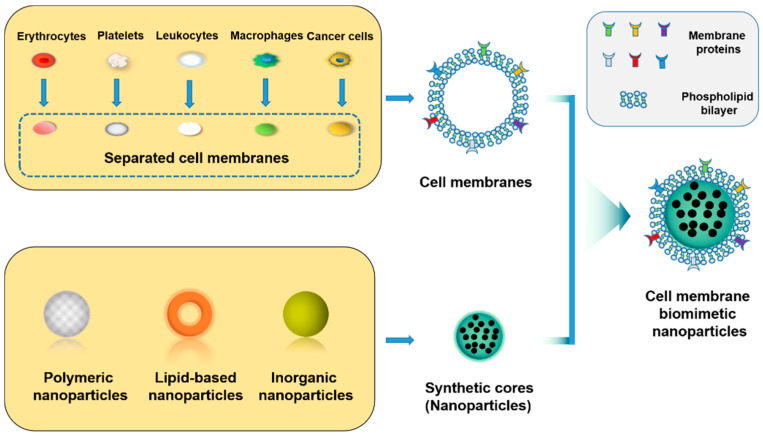
Schematic diagram of the preparation method of cell-membrane-coated NPs.

To solve this problem, many researchers have designed a class of cell membrane biomimetic nanoparticles (NPs) that can cross the BBB to the affected area in the brain. Cell membrane biomimetic NPs typically consist of a thin cytoplasmic membrane and encapsulated therapeutic NPs, resulting in a “core–shell” structure in which drug-encapsulated NPs (polymeric NPs, lipid-based NPs, inorganic NPs) are the “core” [9] and the outer layer of the cell membrane that wraps the NPs is the “shell” (Figure 1).

The “core” of drug-carrying cell membrane biomimetic NPs is prepared from nanomaterials and drugs, which can prolong the drug’s half-life and increase biocompatibility in nerve tissues to enhance the therapeutic effect [10]. To further improve delivery efficiency, the NPs are wrapped with appreciable amounts of natural cell membranes that could be easily resized to form the “shell” of NPs [11]. Cell membrane biomimetic NPs, as a new type of nanomedicine, can target disease sites through the homing tendency of membrane proteins while also evading immune elimination, extending circulation time, and other biological functions of the parent cell [12]. In contrast, “core–shell” cell membrane biomimetic NPs are more complex but more flexible and less repulsive than simple NPs without biomimetic membranes [13]. Core NPs can also be formulated to be loaded with drugs to enhance stability of NPs in blood. Cell membrane biomimetic NPs can also be modified with specific peptides targeting the BBB or diseased sites. Taking advantage of the high transmembrane transport efficiency of targeting peptides, targeting peptides are used as carriers to couple with cell membrane biomimetic NPs to “carry” the cell membrane biomimetic NPs to penetrate the BBB, thereby significantly increasing the efficiency of cell membrane biomimetic NPs and enhancing active targeted delivery of NPs. Biomimetic nanotechnology provides a new idea for designing nanomaterials that cross the BBB, which is expected to be used in treatment of AD [14].

In the following review, we focused on the influence of BBB and application of cell membrane biomimetic NPs in treatment of AD. In addition, NPs synthesis methods, cell membrane extraction methods, and cell membrane biomimetic NPs fusion methods were reviewed in this paper. Finally, targeting peptides commonly used to enhance BBB targeting of biomimetic NPs were summarized, aiming to achieve drug delivery to AD lesion areas.

## 2. BBB Hinders AD Treatment

### 2.1. Current Therapy Strategy

Less successful treatment drugs have been developed to treat AD [15], and most of them can only temporarily relieve symptoms and improve cognitive function. For symptomatic alleviation of AD, the European Medicines Agency (EMA) suggested cholinesterase inhibitors (ChEIs) and *N*-methyl-d-aspartate (NMDA) receptor antagonists [16]. ChEIs, including donepezil, galantamine, tacrine, and rivastigmine, increase acetylcholine levels in the synaptic cleft by inhibiting acetylcholinesterase, cause temporary cognitive enhancement, and are licensed for mild to moderate AD. NMDA receptor antagonists, such as memantine, reduce neurotoxicity of glutamate for symptomatic relief of moderate to severe AD phases [17]. In addition, the Food and Drug Administration (FDA) also approved marketing of a combination therapy (a fixed-dose mixture of donepezil and memantine) [18] and aducanumab besides ChEIs and NMDA receptor antagonists. The fixed-dose mixture of donepezil and memantine showed more significant effectiveness than donepezil alone [19]. Aducanumab is a monoclonal antibody that treats early-stage AD by reducing amyloid deposition and is the first approved therapy targeting amyloid [20]. However, the EMA rejected marketing of aducanumab after finding abnormal swelling or bleeding in brain of minor patients. Although clinical trials of aducanumab are controversial, treatment with aducanumab still offers advantages over existing treatments [21].

### 2.2. The Impact of BBB on Treatment of AD

**Figure 2:**
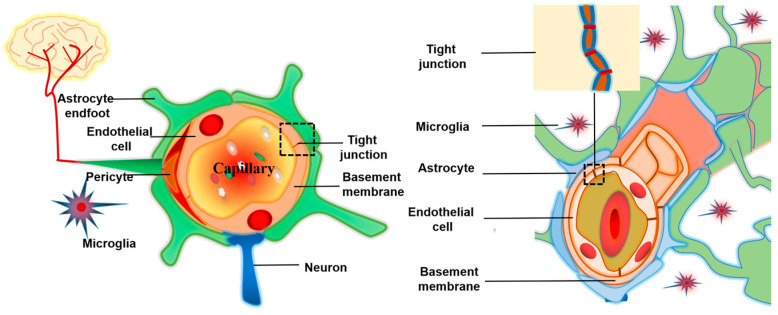
Schematic diagram of the structure of the BBB. The basement membrane surrounds the BCECs and embeds pericytes that span several BCECs. AS end-feet are in contact with the BCECs.

Drug efficiency is enormously limited due to the existence of BBB [22]. BBB is assembled by the blood–brain, cerebrospinal fluid–brain, and blood–cerebrospinal fluid barriers. BBB is composed of brain capillary endothelial cells (BCECs), astrocytes (AS), basement membrane, microglia, tight junctions, neurons, and pericyte, showing high selectivity [23] (Figure 2).

**Figure 3:**
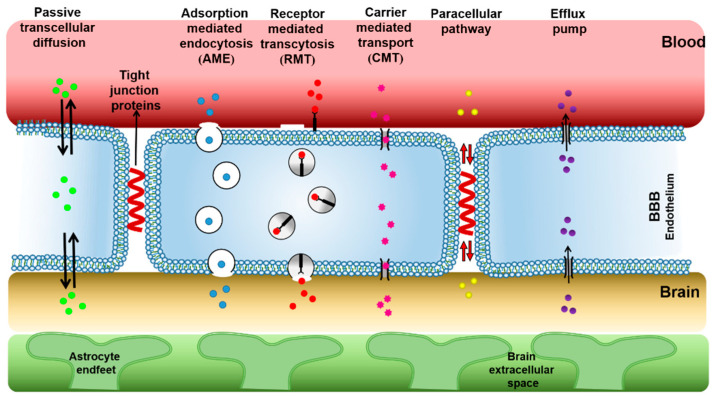
Schematic diagram of the physiological mechanisms by which drugs cross the BBB.

The BBB’s BCECs are surrounded by AS, whose feet processes form connections to neurons. Pericytes are sporadically scattered along the brain capillaries and work with the basement membrane to stabilize the BBB. Microglia are sorts of immune cells exiting the central nervous system. Tight junctions are created by a complex network of parallel, linked transmembrane, and cytoplasmic proteins and are found in the apical/luminal area of BMECs. Only particles smaller than 1 nm are permitted to cross the passage of tight junctions. Thus, the BBB provides a protective mechanism for maintaining brain homeostasis and protecting the central nervous system from harmful hematogenous, endogenous, and exogenous substances. Although drugs or endogenous components cross the BBB into the brain via transcellular pathways [24] (passive transcellular diffusion pathway, paracellular pathway, adsorption mediated endocytosis, carrier mediated transport, receptor mediated transcytosis, and efflux pumps that expel materials from the brain [25], as shown in Figure 3, the transcellular pathways still require certain conditions for molecules to enter the brain, including molecular weight (less than 400 Da), shape (spherical), size (nano range), ionization (physiological pH value), lipophilicity, etc. As a result, all macromolecular drugs and more than 98% of small-molecule drugs are excluded from the brain [26]. Even though many drugs show satisfactory therapeutic effects in vitro, most of the activities are blocked by the BBB in vivo. According to the study of Zhao et al., the bioavailability of donepezil administrated orally in the brain is only 0.024–0.05% [27]. The research of Karasova also revealed that the oral bioavailability of tacrine in the brain is only 0.0014–0.0018% [28]. Presence of BBB has a significant impact on bioavailability of drugs into the brain, which is a great challenge for treatment of AD [29].

### 2.3. New Strategies for Treating AD through BBB

#### 2.3.1. Route of Administration for Treating AD

Faced with the difficulty of delivering drugs into the brain for diagnosis and treatment, there are currently three main approaches to solving this problem: (A) local sustained release, a highly invasive procedure in which a drug is injected directly into the brain through the meninges. However, it is clinically prone to bleeding and central nervous system infection and should be used in very severe cases or only when a patient is hospitalized. (B) Intranasal administration: the brain receives medication directly by bypassing the BBB via the olfactory and trigeminal pathways. However, there are problems, such as insufficient absorption of drugs from the nasal mucosa, small nasal volume, large variability, and poor stability [30]. (C) Systemically administered, where a drug can cross the BBB and arrive in brain when administered orally or intravenously [31]. To date, the systemic delivery route is the most interesting and receptive strategy for AD treatment.

#### 2.3.2. Potential Agents for AD Treatment

In addition to medicines already in use mentioned in Section 2.1, some natural products have shown great potential in treatment of AD, such as curcumin (Cur), quercetin [32], thymoquinone [33], huperzine A [34], and rhynchophylline [35]. At the same time, many potential natural products, such as fucosterol, lectin, fucoxanthin, and astaxanthin, have been isolated from marine algae [36]. Marine actinomyces produce a variety of halogenated compounds and can also be a rich source of natural medicines to treat AD [37]. However, most natural products are poorly water-soluble, resulting in difficulties to enter the brain. NPs could be used as vectors to carry natural products across the BBB to treat AD. Among them, metal NPs can easily be synthesized, coated, or combined to carry these natural products. Metal NPs carrying natural products can be used for in vitro detection of Aβ using confocal microscopy and also to manipulate Aβ aggregation to treat neurodegenerative diseases [38].

#### 2.3.3. Nanoparticle Technology for Treating AD

There are several routes in which nanocarriers carry therapeutic drugs across the BBB into the brain to treat degenerative neurological disease: (A) open the tight connections between BCECs or cause local toxic effects that make the BBB more permeable, allowing NPs to enter the brain; (B) through endocytosis through BCECs, NPs enter the BCECs cytoplasm and then are expelled into the brain by the BCECs lumen; (C) through transcellular action through BCECs into the brain. NPs’ biocompatibility and biodegradability allow them to protect pharmaceuticals, increase their bioavailability, and enable active targeting through surface functionalization, making it easier to distribute drugs to specific places. NPs may be able to transport therapeutic medications directly to the brain due to these advantageous effects without endangering the BBB’s neuroprotective qualities [8]. Therefore, NPs can be used to target amyloid Aβ aggregation, excessive *Tau* phosphorylation, neurotransmitter dysfunction, neuroinflammation, oxidative stress, and neurotrophic factors in the brain to restore degenerative nerves and thus achieve the therapeutic effect of AD. NPs carry fluorescent molecules for early diagnosis of brain diseases. In conclusion, application of NPs in treatment of AD across the BBB has shown great potential.

In the systemic delivery system, cell membrane biomimetic NPs have shown great advantages for brain drug delivery, with the features of high drug loading, good biocompatibility, long circulation time, instinctual targeting ability, and low immunogenicity. Cell membrane biomimetic NPs simultaneously combine the advantages of unique natural cell membranes and an artificial core and can disguise as autologous cells, providing a new idea for brain drug delivery.

## 3. Core NPs

With the introduction of the concept of nanotechnology, nanotechnology has become a field to ensure safety of human life, involving many aspects, such as medicine and bioscience, agriculture, the food industry, electronics, transportation, communication, energy, etc. [39]. NPs consisting of nanomaterials and therapeutic agents [40] have characteristics of tissue selectivity, long circulation time, protecting and encapsulating drugs, enhancing drug absorption, increasing the loading and bioavailability of poorly soluble drugs, etc. [41]. On this basis, a variety of drug nanocarriers have been developed, including polymeric NPs, lipid-based NPs, and inorganic NPs.

### 3.1. NPs

#### 3.1.1. Polymeric NPs

Because of their flexible architecture (10–1000 nm), low toxicity, biocompatibility, and regulated drug release, polymeric NPs—both synthetic and natural—have been extensively exploited in an astounding variety of drug delivery systems [42]. Therapeutic medications can be transported across the BBB by passive or active delivery when they are enclosed in polymeric NPs, protecting them from enzymatic and hydrolytic destruction [43]. According to reports, pharmaceutically loaded polymeric NPs boost brain penetration, enabling much higher drug concentrations at the target region and increasing the medicine’s overall effectiveness [44].

The most common biodegradable and biocompatible polymers used to create polymeric NPs include poly(d,l-lactide-*co*-glycolide) (PLGA), polyethylene glycol, polylactic acid, poly(caprolactone), poly(glutamic acid), *N*-(2-hydroxypropyl)-methacrylate copolymers, poly(*n*-butylcyanoacrylate), 4-(Hydroxymethyl) phenylboronic acid pinacol ester (PBAP), and poly(amino acids), etc. PLGA is a synthetic polymer that has received FDA approval and has excellent biocompatibility and biodegradability features. It has been utilized extensively in medicinal applications, including treatment of AD [45].

Gao et al. prepared Cur-encapsulated PLGA NPs and camouflaged them with a red blood cell membrane (RBCm) (RBC-NP-Cur). In aneuronal-like cells (HT22) and primary brain capillary endothelial cell Transwell co-culture test, RBC-NP-Cur was confirmed to show significantly better permeability than Cur. In addition, after injection of RBC-NP-Cur in AD model mice, more accumulation of Cur, reduced cognitive decline, suppressed nerve cell death, and decreased *p*-tau levels in the brain were observed compared to free Cur group [46]. Tang et al. used macrophage membrane (MA)-encapsulated rapamycin-loaded PBAP NPs to treat AD. Confocal laser scanning microscope images revealed that a significant number of MA/1,19-Dioctadecyl-3,3,39,39-tetramethylindo-dicarbocyanine perchlorate (DiD) NPs accumulated in inflammatory human umbilical vein endothelial cells (HUVECs) after NPs were modified with DiD for 2 h [47]. These results imply that modified polymer NPs can actively target the sick region and release medications, resulting in increased effectiveness of drug delivery.

Polymer nanomicelles (PM) carrying a wide range of proteins, such as mRNAs or antibodies, can specifically and efficiently penetrate the BBB by injection to reach the brain parenchyma to release active agents’ fragments. In addition to preventing early degradation of mRNA, use of mRNA-loaded PM altered intracellular mRNA transport, increased mRNA detection and expression in neurons in vivo, and reduced amyloid load in mouse models of acute amyloidosis [48]. It was also shown that accumulation of 3D6 antibody fragments (3D6-Fab) in the brain using the PM system was 41 times greater than that of free 3D6-Fab and successfully inhibited Aβ_1-42_ aggregation in AD mice [49]. According to the characteristics of high impermeability of BBB, biotin and PECAM-1 protein on BBB were combined to form biotin targets on BCECs and avidin-functionalized PM with biotin specific binding were prepared. The PM can only target the brain and reduce accumulation in other organs in the body, thus reducing clinically limited peripheral side effects and effectively treating AD [50].

#### 3.1.2. Lipid-Based NPs

Liposomes [51], solid lipid NPs (SLNs) [52], nanostructured lipid carriers [53], and nanoemulsions [54], among others [32], are the most common lipid-based NPs. Phospholipids, fatty acids, or cholesterol are generally used to create lipid-based NPs. Lipid-based NPs have the ability to form both unilamellar and multilamellar vesicular structures with diameters between 80 nm and 100 μm. Lipid-based NPs can carry various kinds of therapeutic drugs (such as nucleic acids, enzymes, and proteins) for effective transportation. The unique phospholipid structure of lipid-based NPs (similar to physiological membranes) makes them more compatible with the cellular lipid layer of the BBB, facilitating drug entry into the brain. Controlled and exact release of the materials at the targets is also made possible by lipid-based NPs, which can limit the rate of material breakdown. Lipid-based NPs are widely used in brain-targeted drug delivery because of high safety, good economic performance, large drug loading, good biodegradability, and biocompatibility [55].

Song et al. encapsulated rapamycin in liposomes and coated with platelet membrane by extrusion (P-Lipo). After intravenous injection of P-Lipo, the extracted brain tissue was observed under a fluorescence microscope and it was found that accumulation of P-Lipo in atherosclerotic lesions increased by 5.91 times compared with control liposomes, indicating that P-Lipo-specific targets to atherosclerotic plaques [56]. Han et al. prepared MA-coated SLNs for delivery of genistein to neuronal mitochondria (MASLNs). Using the brain microvascular cell (bEnd.3)/HT22 in vitro co-culture model to study the permeability of the BBB, it was found that MASLNs had a more pronounced ability to cross the BBB than SLNs. In vivo imaging in mice following tail vein injection of 1,1′-dioctadecyl-3,3,3′,3′-tetramethylindotricarbocyanine iodide (DiR)-labeled MASLNs and fluorescence intensities identified in isolated brain homogenates revealed that MASLNs can cross the BBB [57].

#### 3.1.3. Inorganic NPs

Inorganic NPs are usually based on metals and non-carbon sources. The shape and size of inorganic NPs, including gold (Au) [58], silica (Si) [59], silver (Ag) [60], and magnetic (Fe_3_O_4_) [61] NPs, can be precisely tuned according to the synthesis process. Inorganic NPs have large surface area, controllable structure, diverse surface chemical properties, unique optical and magnetic properties, etc. At the same time, inorganic NPs also have chemical and physical stability in treatment of brain diseases and diagnostic applications [62]. Additionally, use of particular external stimuli, such as magnetic materials and near-infrared light, can assist the BBB’s on-demand drug release and improve imaging [63]. However, because of their inherent toxicity, inorganic NPs are not always simply removed from the body, can have long-lasting immunological reactions, and have low biocompatibility [63].

Plissonneau et al. prepared sub-5 nm gadolinium-based NPs (AGuIX) co-modified by two small peptides derived from the sequence of Aβ_1–42_, KLVFF and LPFFD. KLVFF matches the short hydrophobic core Aβ segment and LPFFD binds the hydrophobic area in Aβ’s center, respectively, for Aβ-amyloid-plaque-targeted treatment of AD. Both functionalized NPs attach specifically to the amyloid plaque made up of Aβ protein in vitro hippocampus of AD model mice, as shown by immunohistochemical labeling with Pittsburgh compound on brain slices of transgenic mice with AD [64]. Gao et al. prepared an Au NPs scaffold with both polyoxometalates with LPFFD and Wells–Dawson structure (POMD) (AuNPs@POMD-pep). Thioflavin T assay demonstrated that AuNPs@POMD-pep successfully promoted disintegration of Aβ fibrils after co-incubating Aβ monomers with the compound. This was demonstrated by the fact that the fluorescence intensity was reduced by roughly 37%. After injection of AuNPs@POMD-pep to mice intravenously, inductively coupled plasma mass spectrometry analysis was involved to verify the success penetration of AuNPs@POMD-pep across the BBB [58].

### 3.2. Synthesis of Core NPs

#### 3.2.1. Single Emulsification–Solvent Evaporation Method

**Figure 4:**
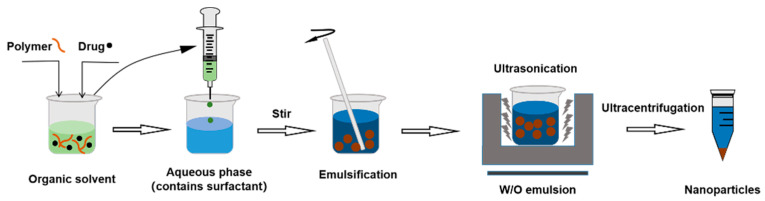
The procedure of single emulsification–solvent evaporation method.

Single emulsion (W/O) of polymeric NPs for hydrophobic drugs can be synthesized by emulsification–solvent evaporation method. First, the nano carries and interested ingredients are dissolved in organic solvents as organic phase. The organic phase is dropped into water phase containing sodium cholate, poloxamer, etc. Then, the mixtures are stirred and phaco-emulsified to produce a stable nano-emulsion. The organic solvent is evaporated off to generate NPs [65] (Figure 4).

#### 3.2.2. Double Emulsion Method

**Figure 5:**
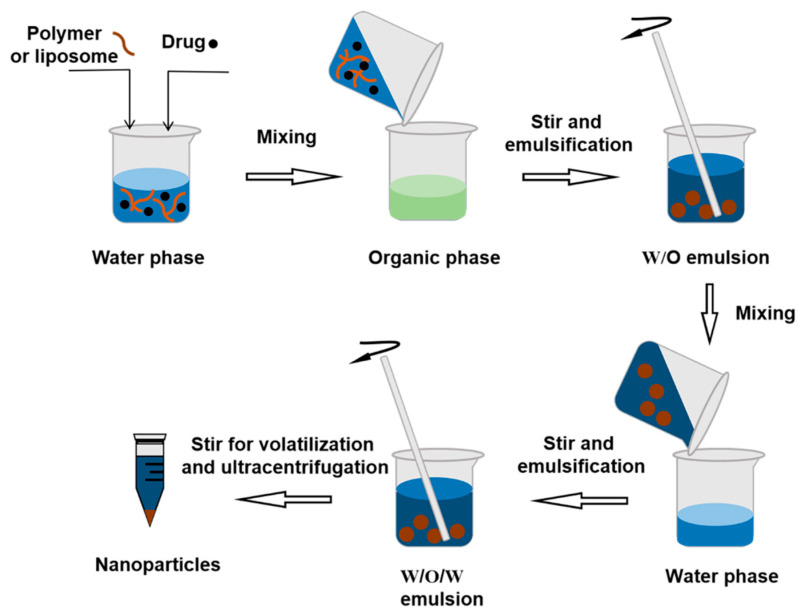
The procedure of double emulsion method.

Generally, hydrophilic drugs (such as proteins) can be prepared as polymeric NPs and lipid-based NPs by this double emulsion (W/O/W) method [66]. The hydrophilic drug and stabilizer are first dissolved in water and then dispersed in the organic solvent form colostrum. The colostrum is dispersed and dissolved in an aqueous phase containing an emulsifier, and the formulation is finally stirred to evaporate the organic solvent and obtain NPs [67]. The double emulsification method can prevent the decrease in encapsulation efficiency caused by the rapid dispersion of water-soluble drugs into the outer water phase during the emulsification process (Figure 5).

#### 3.2.3. Nanoprecipitation Method

**Figure 6:**
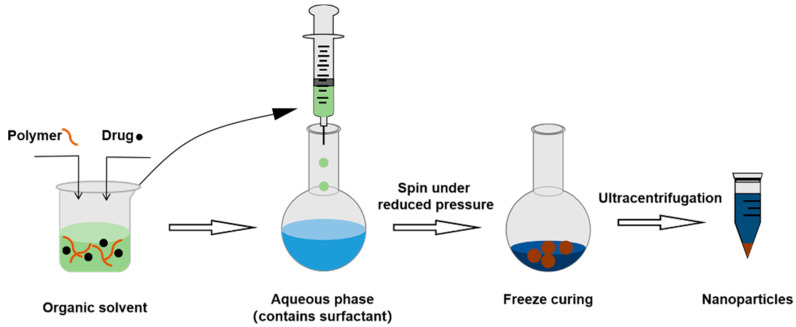
The procedure of nanoprecipitation method.

Nanoprecipitation method can be used to prepare hydrophobic polymers into NPs. Materials and drugs are dissolved and dispersed in organic solvents. The resulting organic solvent is added into an aqueous solution with surfactants magnetically stirred. Due to the difference in the solubility of hydrophobic polymers in the two solvents, the polymers that exist in stretched form in organic solvents will aggregate and form NPs once they enter the aqueous phase. By using lower pressure evaporation or nitrogen blowing, the solvent and water from the particle dispersions are eliminated. NPs are precipitated from the suspo-emulsion by centrifugation in a differential centrifuge [68] (Figure 6).

#### 3.2.4. Salting out Emulsification–Diffusion Method

**Figure 7:**
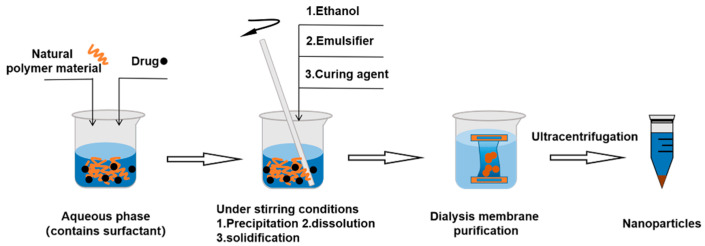
The procedure of salting out emulsification–diffusion method.

No toxic organic solvent is used in salting out emulsification–diffusion method. Natural polymer materials, such as albumin and gelatin, are used as carrier materials to prepare polymeric NPs. Dissolve nanocarriers and hydrophobic drugs in water with surfactant, and then add salt precipitating agent or change the pH value to precipitate the polymer while stirring. An emulsifier is added to emulsify the precipitate and an appropriate amount of curing agent is used to stabilize the NPs. Dialysis membrane or gel column chromatography can be used to purify the products [69] (Figure 7).

#### 3.2.5. Supercritical Fluid Method

**Figure 8:**
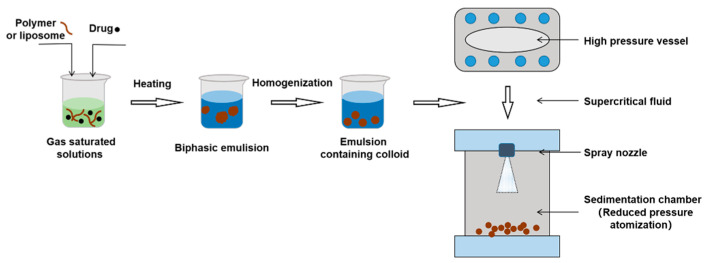
The procedure of supercritical fluid technology.

Supercritical fluid is a substance in a supercritical state that can improve solubility of poorly soluble drugs [70]. Polymers (or liposomes) and drugs are dissolved in a supercritical liquid. When the supercritical liquid is decompressed and atomized through a small-diameter nozzle, the NPs will be separated with rapid vaporization of the supercritical liquid [71]. Supercritical fluid technology is emerging as an attractive preparation method due to use of environmentally friendly solvents, high-purity processing of NPs, and absence of residual organic solvents [72] (Figure 8).

#### 3.2.6. Spray Drying Method

**Figure 9:**
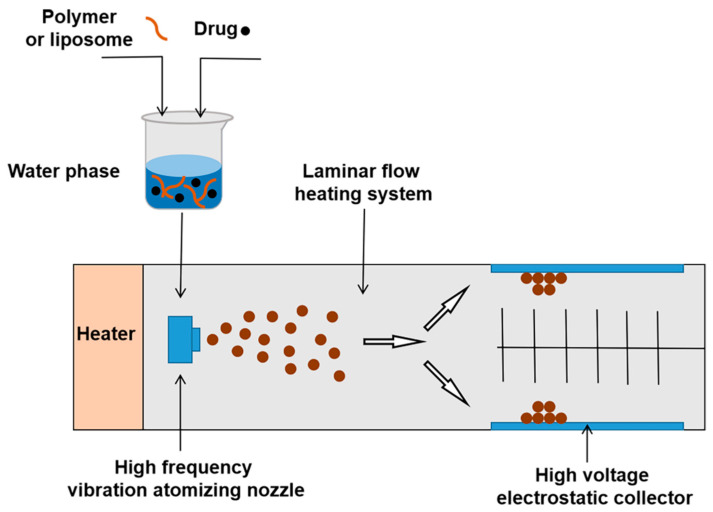
The procedure of spray drying method.

Spray drying method has been shown to be an effective way for improving long-term stability of NPs and is suitable for industrial largescale preparation of NPs. With removal of water, NPs material turned from fluid state to powdered material. The atomizer atomizes a certain concentration of fluid, falls into a certain flow of dry gas for vaporization/drying, and finally separates NPs from the dry gas [73]. This method is further restricted to lipids with a melting point over 70 °C (Figure 9).

#### 3.2.7. Solvothermal Method

**Figure 10:**
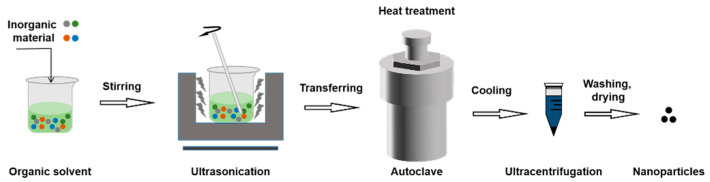
The procedure of solvothermal method.

In closed reactors (such as autoclave) with high temperatures and pressures, the solvothermal approach typically functioned and is suitable for preparation of inorganic NPs [74]. The raw material is dissolved in an organic solvent, stirred, sonicated, and then transferred to an autoclave for heat treatment. Then, the cores evolve into grains with a certain shape. The autoclave spontaneously cools down to ambient temperature in the presence of air following the solvothermal reaction [75]. The precipitate is then centrifuged separately, cleaned with deionized water, and finally dried [76]. The reaction process is relatively sluggish with this approach, taking between 18 and 36 h [77] (Figure 10).

#### 3.2.8. Sol–Gel Method

**Figure 11:**
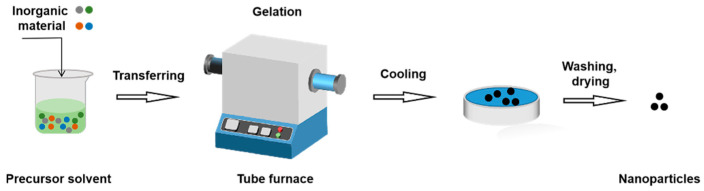
The procedure of sol–gel method.

When using the sol–gel process, the inorganic material is uniformly dissolved in the precursor solvent before being transferred to a container. Heat the container, store under vacuum for a period of time and cause its gelation, and then cool to room temperature. The product is washed with ethanol and subsequently dried under vacuum to obtain inorganic NPs with core–shell structure. By adjusting the reaction time and material ratio as well as the proportion of the reaction product, the sol–gel method can control the thickness of the shell, which in turn affects the overall particle size [78] (Figure 11).

#### 3.2.9. Thermal Decomposition

**Figure 12:**
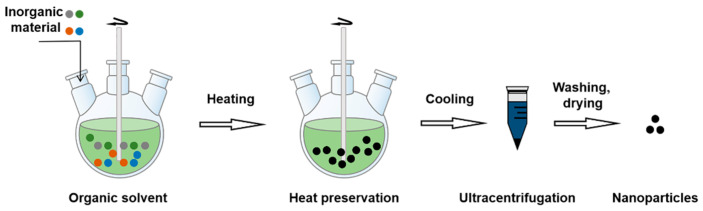
The procedure of thermal decomposition.

The weighed precursor and inorganic material are added into an organic solvent. The mixture is stirred to dissolve during heating and then kept at a specific temperature in the reactor. The samples are allowed to cool down at room temperature. Products are collected by filtration, washed several times, and dried overnight [79]. Thermal decomposition has the benefit of allowing for efficient and speedy synthesis of functional NPs in accordance with experimental requirements (Figure 12).

## 4. Cell Membrane

As the “shell” of the cell membrane biomimetic NPs, the cell membrane plays the role of modifying the NPs. Membrane proteins embedded in semipermeable phospholipid bilayers, such as integrins, peripherins, and lipid-anchored proteins, make up around one-third of all the proteins in an organism [80]. NPs, wrapped in membranes that embed endogenous proteins outwards, would have the properties of endogenous cells, enhancing immune escape and extending blood circulation [81]. This chapter summarized the cell extraction methods from blood or tissue and their corresponding membrane properties and limitations (as shown in Table 1).

### 4.1. Source Cell

Distinct types of cells in the human body perform several physiological tasks, such as lengthy blood circulation, migration to particular body areas, and traversing physical barriers. To deliver medications with retained cellular structure and function, it is crucial to choose particular cell types.

#### 4.1.1. Erythrocyte

Erythrocytes, also known as red blood cells, lack organelles and nuclei and are biconcave blood cells, with a diameter of between 7 and 8 μm and a central thickness of about 1 μm. Erythrocytes are the most prevalent cell in blood, making up a quarter of the total number of cells in the body, and can be easily isolated from the blood [82]. In addition, because of a lifespan of more than 120 days and high surface area/volume ratio, the erythrocyte has long circulation time in the body for efficient transport. Erythrocytes can effectively avoid phagocytosis by the immune system because the surface of erythrocytes is rich in self-labeled proteins, such as CD47, polysaccharides, and acidic sialic acid moieties, which are essential for maintaining balance of erythrocytes [83]. Deactivation of myosin IIA, which is involved in contraction of macrophage actinomyosin, is brought about by binding of CD47 to SIRP-α and can prevent phagocytosis [84]. In addition to camouflaging their function and avoiding clearance by the immune system, NPs modified with RBCm can reduce toxic side effects, prolong circulation time, and enhance drug retention at the focal site [85]. In recent years, polymeric NPs encapsulated by RBCm have represented an emerging nano-drug delivery platform because of their properties of prolonging circulation in vivo [86].

Gao et al. coated Cur-loaded human serum albumin NPs with RBCm and then modified the RBCm surface with triphenylphosphine (TPP) molecules and AV-1451 (T807) (T807/TPP-RBC-Cur-NPs). In the in vitro co-culture model of BMECs, AS, and HT22 cells, significant fluorescence intensity was observed in the T807/TPP-RBC-NPs group, proving that RBCm-coated biomimetic NPs could effectively traverse the BBB and fully enhance the Cur cell uptake. Hematoxylin and eosin staining was used to check the survival of neurons in the hippocampus of AD model mice after injection of T807/TPP-RBC-Cur-NPs into the tail vein. The neuronal survival rate in the hippocampus of AD model mice treated with T807/TPP-RBC-Cur-NPs was greatly increased [87].

#### 4.1.2. Platelet

Platelets are disc-shaped anucleate cellular fragments from bone marrow stem cell line megakaryocytes [88]. Platelets are less numerous (150,000-450,000/mL), shorter lifespan (7–10 days) [89], and smaller (average diameter 2–4 μm) compared to erythrocytes [88]. Platelets are commonly used for vascular injury, wound healing, inflammation, and hemostasis following thrombosis [90]. Due to the ability of platelets regarding immune evasion [91], subendothelial adhesion, and pathogen detection [92], platelet-membrane-coated NPs have gained great attention over the past few decades.

Xu et al. synthesized YGRKKRRQRRR-NH_2_-modified platelet membranes coated with neuroprotectant (ZL006e) and recombinant-tissue-plasminogen-activator-loaded polymeric NPs of glucan derivatives (TP-NP-rtPA/ZL006e). It was found that the cell penetration rate of TP-NP-rtPA/ZL006e in BCECs cells was significantly higher than that of free ZL006e. The concentration of TP-NP-rtPA/ZL006e in the brain was 14.6% after injection of coumarin-6-labeled TP-NP-rtPA/ZL006e into a mouse model, which was higher than that in liver tissue at 2 h administration. TP-NP-rtPA/ZL006e was found to penetrate the BBB and accumulate in injured neural tissue [93].

#### 4.1.3. Leukocyte

Leukocytes, commonly termed as white blood cells, are between 7 and 20 μm in diameter, larger than erythrocytes due to their nucleus. Leukocytes play an important function in protecting bodies from infection and injury and are widely distributed in blood arteries, lymphatics, and other organs. The CD11b protein on the surface of leukocyte membranes can target activated BCECs and evade uptake by the mononuclear phagocytosis system via CD45 protein [94]. Based on the characteristics of receptor proteins on the leukocytes membrane, the leukocytes membrane can be used as a general tool for therapeutic drug delivery [95]. Leukocytes come in five different subtypes: neutrophils (NCM), eosinophils, basophils, lymphocytes, and monocytes. Leukocytes are the guardians of the body, with unique physicochemical and biological properties. Therefore, NPs disguised with leukocyte membranes have the effect of evading opsonization and delaying uptake [96]. As the existence of nuclei, leukocytes are more difficult to be extracted and purified. Despite of their flaws, white blood cell membranes also are excellent candidates for NPs encapsulation due to their capacity to target and regulate tumors and inflammation.

Mesoporous Prussian blue nanozyme, which has a cell membrane coating of NCM (MPBzyme@NCM), was created by Feng et al. After co-culture with MPBzyme@NCM and Transwell system (bEnd.3 and mouse microglia cells), it was found that MPBzyme@NCM could not only cross BBB but also had strong reactive oxygen species scavenging ability. Fluorescence imaging of mice injected with fluorescein isothiocyanate labeled MPBzyme@NCM showed that NCM coating promoted continuous accumulation of MPBzyme@NCM in brain tissue compared with NCM or non-coating [97].

#### 4.1.4. Macrophages

Macrophages are immune cells that recognize, engulf, and digest cellular debris and other foreign substances that lack biomarkers of their own [98]. Recently, macrophages have attracted much interest and have been developed to contain NPs [99]. As a kind of immune cell, macrophages have the function of immune evasion [98]. NPs that have been enclosed by the MA can easily move between macrophages, extravascular tissues, and blood vessels. Studies have shown that pathogeny of AD is related to damage of BCECs [100], which secrete adhesion molecules caused by immune cells. Thus, macrophages could be transferred to the lesion site and participate in various stages of AD [101].

Long et al. prepared Baicalin liposomes (BA-LP) modified with MA (MA-BA-LP). Intensity and duration of fluorescence in brains of mice injected with MA-DiR-LP in tail veins were substantially higher than those in the DiR-LP group. The findings demonstrated that MA-BA-LP had a greater brain targeting impact than BA-LP [102].

#### 4.1.5. Cancer Cells

Cancer-cells-modified NPs can be easily bound to homologous tumor cells because of the homologous adhesion molecules on cell membranes, such as lectins, integrins, cadherins, selectors, and proteins [103]. Cancer-cell-membrane-modified NPs have strong homologous targeting capabilities without the need for laborious surface modifications, and they can be employed to deliver medications or contrast agents to specific tumors [104]. As a result, NPs coated with cancer cell membrane also can improve cellular uptake, tumor targeting, and accumulation in addition to enabling endogenous biomimetic “stealth” administration in vivo [105]. Furthermore, it has been established that one of the best strategies to improve NPs’ ability to be biocompatible for treatment of homologous malignancies is through cancer cell membrane. Studies have shown that NPs disguised as glioma cell membranes have the ability to cross the BBB and long-term circulation [11], but, unfortunately, treatment of AD with cancer-cell-membrane-modified NPs is unclear [106].

#### 4.1.6. Membrane Hybridization

Multi-membrane-modified NPs showed better performance compared with single-membrane-modified NPs [107]. Membrane hybridization can introduce membrane proteins specific to one cell type into another, giving the hybrid membrane greater targeting ability. Membrane hybridization can also enhance the immune escape of NPs by introducing another membrane with stronger stealth ability. Membrane hybridization is also used to introduce “homologous” characteristics that reduce unnecessary cells [108]. Mixed membranes exhibit superior biocompatibility, reduced immunogenicity, prolonged cyclicity, homologous targeting capability, and stimulation of the innate or adaptive immune system. For instance, modification of NPs by fusing of platelets and other functional membranes allows them to target damaged areas and treat diseases such as atherosclerosis and acute inflammation. Bionic neutral granulocytes and macroscopic cells with collaborative tumor micro-environment modify nano-particles to treat homologous tumors [109].

#### 4.1.7. Other Cells

In a particular design found in the study, Niu et al. attached doxorubicin-loaded heparin NPs to the surface of native grapefruit extracellular vesicles, creating biomimetic grapefruit for drug delivery NPs; compared with traditional cell membrane encapsulation, the therapeutic effect of modified NPs can reach 4 times the drug loading. Biomimetic grapefruit NPs can bypass BBB and enter glioma tissue through receptor-mediated cell penetration and membrane fusion, which greatly promotes cellular internalization and anti-proliferation capabilities and extends the cycle time [110].

### 4.2. Isolation of Cell Membrane

**Figure 13:**
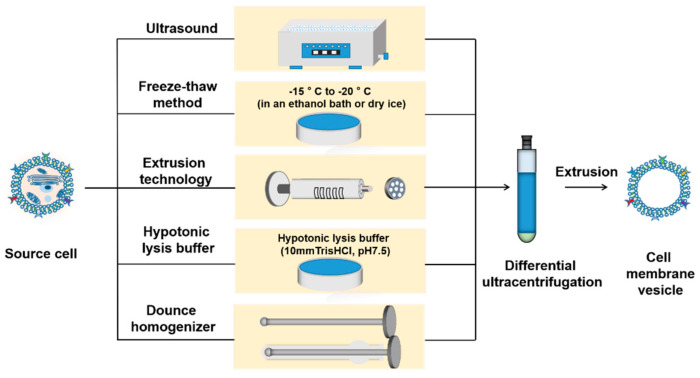
Schematic representation of different methods for isolating cell membranes from native cells.

Natural, intact, and functional cell membranes are separated from source cells by destroying or lysing cells to empty their intracellular components. Cell membranes can be separated from blood or tissues by ultrasound, freeze–thaw, extrusion, hypotonicity, and Dounce homogenizers (summarized in Figure 13).

#### 4.2.1. Ultrasound

Ultrasound is a technique that agitates cells and their constituent parts with sound energy. The tube containing the cell suspension is placed in an ultrasonic bath [111]. The alternating process of compression and expansion through different acoustic frequencies produces intense shock waves (inertial cavitation) [112] and high-velocity microjets [113], known as cavitation. The cavitation leads to cell rupture and cell lysis, which makes it easier to separate the cell membranes. However, ultrasonic membrane separation technology still has some shortcomings. For example, free radicals generated in the process of ultrasound can affect the internal structure of cell membrane. The increasing temperature of the medium can change the properties of membrane proteins, resulting in unstable yield of cell membrane [80]. To prevent permanent harm to cell membranes, the power, frequency, time, and temperature parameters should be carefully optimized.

#### 4.2.2. Freeze–Thaw

Freeze–thaw method is a gentle, simple, and convenient method of cell membrane isolation. The culture dish holding the cell suspension is quickly thawed at 40 °C or at ambient temperature after being instantly frozen at −15 °C to −20 °C (either dry ice or an ethanol bath). Cell destruction occurs after at least two cycles of freezing and thawing [114]. This is because cells grow larger by forming frozen ice crystals, which then contract as they melt. When the sizes of the ice crystals cells are periodically changed, the cells rupture and the membranes are separated. During freezing and thawing cycling, proteins embedded in the cell membrane are damaged and protein activity will be inhibited.

#### 4.2.3. Extrusion

Cell membranes with large cross-sectional area are extruded through a die to obtain cell vesicles with smaller cross-section. After pretreatment of membranes with large cross-sectional areas, the cell membrane is mechanically pushed through a mold with a specified cross-section to obtain cell membrane vesicles of a certain desired shape and size [115]. The cell suspension is pushed through a membrane filter on the device using a liposome extruder to obtain cell vesicles in specified size range [116]. The main elements influencing the size distribution of the extruded material are pushing pressure, pushing timings, and pore size of the filter membrane. Extrusion can be conducted at room temperature or above the recrystallization temperature of the material.

#### 4.2.4. Hypotonicity

When the hypotonic lysate is added to the culture dish containing the cells, the water molecules in the hypotonic lysate will diffuse into the cells and increase the volume of the cytoplasm, resulting in rupture of the cells to acquire the cell membrane [117]. Alternatively, cells are suspended in an ice bath in hypotonic lysis buffer for 15 min and then homogenized to obtain a supernatant containing cell membrane fragments and cytoplasmic extract. The pH and osmotic pressure of the lysate are regulated by buffer salts and ionic salts. The ready-to-use, detergent-free hypotonic lysis buffer (10 mm Tris HCl, pH 7.5) has a noticeable impact on cell lysis and cell membrane isolation; 0.25% PBS or 0.4% NaCl [80] is also recommended as a hypotonic lysis buffer for cell membrane isolation.

#### 4.2.5. Dounce Homogenizer

Homogenization is a process of micronizing and homogenizing dispersion in a suspension (or emulsion) system. The Dounce homogenizer [118] is a cylindrical glass tube with two grinding rods of different sizes in each set. The smaller rod for preliminary tissue separation and the larger rod for finishing grinding are appropriate for cell suspension or soft tissue grinding, moderate homogenization of eukaryotic cells, and organelle separation [119]. The cells are placed in a Dounce homogenizer with the appropriate volume of lysis buffer. They are first tapped with a loose rod, and then a large and tight stick is tapped up and down in the glass bottle, usually five to ten times, to obtain the disrupted cell membranes. Previous studies showed that the combination of Dounce homogenizer and hypotonic lysis buffer can achieve better cell lysis effect [120]. Use of a hypotonic buffer caused the cytoplasm to swell, and then the cell membrane is obtained by gently breaking the cell by mechanical force using a Dounce homogenizer. Because the spherical design of the pestle head effectively reduces heat generation during friction, the Dounce homogenizer can avoid the effect of heat accumulation on the protein activity on the cell membrane surface.

### 4.3. Fusion of Membrane Vesicles and NPs

**Figure 14:**
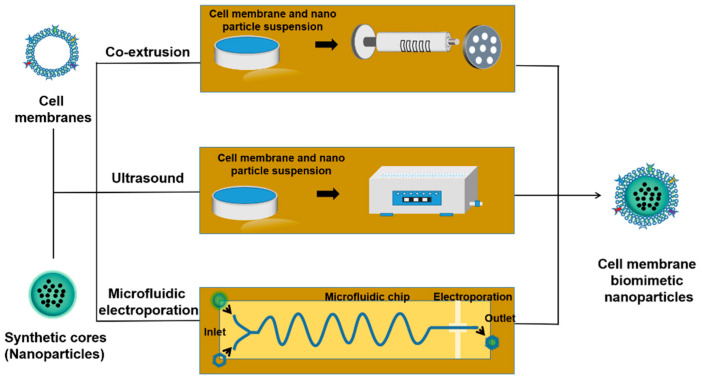
Schematic representation of different methods for fusing cell membranes with NPs.

After the cell membrane is extracted, the extracted cell membrane vesicles need to be fused with NPs to form cell membrane biomimetic NPs. Several methods have been proposed to fuse cell membranes with NPs, including co-extrusion (by mechanical extrusion), ultrasound (endocytosis by ultrasonic energy), extrusion/microfluidic electroporation (by electrical pulses), etc. (Figure 14).

#### 4.3.1. Co-Extrusion

In the co-extrusion method, the cell membrane suspension and the NPs suspension are mixed in certain proportion. The mixed suspension is co-extruded multiple times through a porous filter membrane of the specified size of the extruder [102]. After extruder, NPs are wrapped by cell membrane vesicles to form cell membrane biomimetic NPs. The excess cell membrane vesicles and NPs are discarded by centrifugation. The lower residues after centrifugation are retained as cell membrane biomimetic NPs [116]. Co-extrusion method is easy and simple, and the desired size of cell membrane NPs can be obtained by adjusting the pore size of the filter membrane [121].

#### 4.3.2. Ultrasound

The mixture of cell membranes and core NPs is incubated under the effect of ultrasound to obtain cell membrane biomimetic NPs [122]. The energy generated by the ultrasonic wave is destructive, which can make the cell membrane spontaneously remodel on the NPs to obtain cell membrane biomimetic NPs. Compared with the co-extrusion method, the ultrasonic method has less material loss and simple operation, which shows better application prospects. The ultrasonic method can also fuse multiple membranes to form membrane hybrids, giving the cell membrane more targeting capabilities. However, the film coating formed by ultrasonic method may not be uniform, and the long-term ultrasonic process will also break the NPs.

#### 4.3.3. Microfluidic Electroporation

Microfluidic electroporation technology is a new technology developed in recent years that simultaneously solves the problems of destruction of NPs via ultrasonic/extrusion and time-consuming and labor-intensive nature of the co-extrusion method [123]. When the cell membrane suspension and the NPs suspension are separately injected into the instrument, the two components will first be combined in the first channel (Y shape), then mixed in the second channel (S shape). Under electrical impulses in the perforated area, NPs will penetrate into the cell membrane vesicles, and, finally, the cell membrane biomimetic NPs will be collected from the chip [124]. Even though the synthesis process is relatively complex, this method has high synthesis rate and good parallelism with great potential [125].

#### 4.3.4. Other Coating Methods

In addition to the three methods summarized in Table 2, methods such as freeze–thaw/ultrasound, extrusion/ultrasound and stirring, extrusion/electroporation, and in situ packaging can also be used to prepare cell membrane biomimetic NPs.

## 5. Targeting Peptides

Nowadays, application of membrane biomimetic NPs is becoming more and more common, and the multifunctional demand for membrane biomimetic NPs is also increasing. On this basis, peptides with additional functions can be used, which provide the particles with various additional functions beyond the natural functions of the cell membrane, greatly increasing application of NPs. The usual method includes (A) lipid insertion: ligand–joint–lipid junctions are first synthesized and then the lipid chains are inserted into the membrane depending on the fluidity of the bilayer lipid membrane [126]; (B) membrane hybridization: by fusing a variety of cell membranes, the combined hybrid membrane will have a variety of cell membrane functions [83]; (C) metabolic engineering: metabolic substrates are first bound to functional parts and then incubated with cells to participate in the absorption and metabolic processes, and metabolic engineering methods are expressed on cell membranes [127]; and (D) genetic modification: genetically modified membranes are created by selectively gene-editing cells, which are then coated with NPs [128]. The most widely used and easiest way to apply is to prepare dual-function joints Malayside-polyethylene glycol-active fat chemically modified living cells. Functional chains are attached to the ends of specific targeting peptides, giving the native cell membrane more function when the cell membrane is intact [129]. As shown in Table 3, a variety of targeting peptides have been applied to treat brain diseases through BBB.

**Figure 15:**
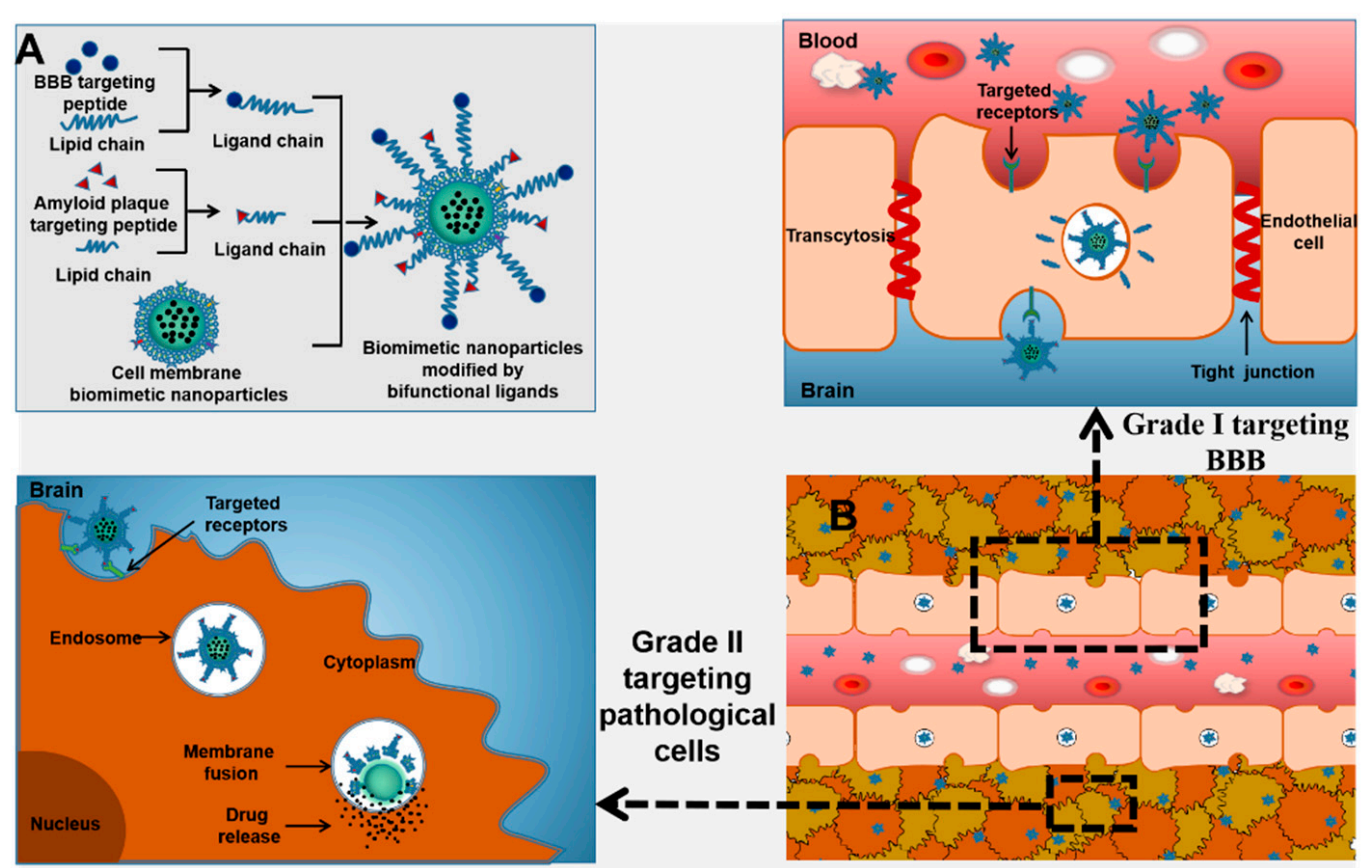
(**A**) Preparation of biomimetic NPs modified by targeting peptides. (**B**) Schematic illustration of the possible mechanism of stepwise targeting of cell membrane biomimetic NPs modified with targeting peptides to the affected area through the BBB for treatment.

On this basis, bi-functional ligands can also be used to modify the cell membrane surface (Figure 15).

## 6. Concluding Remarks and Future Perspectives

Nanomaterials have been developed to treat AD based on promotion of drug delivery across the BBB to achieve accumulation at desired sites. NPs carry therapeutic drugs by encapsulation or surface modification, which has the advantage of effectively delivering drugs to the target area and enhancing the therapeutic effect. Cell membrane biomimetic NPs, which fuse NPs with the membrane of the extracted cell, have the main biological traits and additional physiological functions of the source cell. Cell membrane biomimetic NPs are shielded from the body’s immune system, which prevents immune cells from being absorbed. Surface-modified targeting peptides on cell membrane biomimetic NPs can improve the targeting capabilities and precision to a damaged location.

At present, biomimetic nanomedicine, represented by cell membrane biomimetic NPs used to treat AD, is still in its infancy and has many problems that need to be improved upon. First, maintaining stability of cell membrane biomimetic NPs is one of the issues that must be resolved for application, which requires certain stability of drugs for long-term effective preservation. Delivery efficiency of cell membrane biomimetic NPs is greatly influenced by their size, shape, elasticity, and other physical and chemical properties. Additionally, therapeutic and diagnostic nanomedicine products are currently administered via parenteral injection, typically intravenously. Oral administration is an ideal administration, with high patient compliance. Due to low absorption of solid NPs and biochemical barriers in the gastrointestinal tract, oral delivery of NPs has not proven feasible. Therefore, effective implementation of cell membrane biomimetic NPs administered orally will represent significant advancement in the field of nanomedicine.

## Figures and Tables

**Table 1:** Extraction methods of different cell membranes and their characteristics and limitations.

Cell	Separation Methods	Properties	Limitations
Erythrocyte	Extrusion, ultrasound, freeze–thaw, and hypotonicity	Easy availability.	Poor targeting ability.
		Long circulatory lifespan (~120 days in humans and ~50 days in mice) and wide circulation range.	
		Uniform in size and shape, with a good surface area to volume ratio, without organelles and any DNA.	
		Good biocompatibility, biodegradability, and non-immunogenicity.	
Platelet	Extrusion, freeze–thaw, and ultrasound	High targeting efficiency.	Small proportion of blood and undesirable activated.
		Controlled drug release.	
		Lower immunogenicity.	
		Long systemic circulation (around 7–10 days).	
		Targeting to plaque.	
Leukocyte	Extrusion and hypotonicity	Adhesion capacity.	Organization residency restrictions.
		Migratory and chemotactic capacity in disease states.	
		High loading capacity.	
Macrophage	Extrusion and hypotonicity	Good targeting ability to AD lesions.	Organization residency restrictions.
		Innate immune evasion ability.	
		Long circulation ability in vivo.	
Cancer cell	Extrusion and Dounce homogenizer	Strong homologous targeting ability.	Homologous tumor targeting.

**Table 2:** Methods of fusion of cell membrane and NPs and their advantages and disadvantages.

Method	Procedures	Advantages	Disadvantages
Co-extrusion	The mixed solution formed by mixing the cell membrane suspension and the NPs suspension is co-extruded through a porous filter membrane of specified size with an extruder for many times	The steps are simple and easy to use.	Time-consuming and labor-intensive. Low synthesis rate
		The multi-layer target product can be prepared	
Ultrasound	The mixture formed by mixing the cell membrane suspension and the NPs suspension is sonicated at a certain frequency for a specified time	Less loss of raw materials; mass production is possible.	Uneven coating, easy to form polydisperse particles. NPs are easily broken
		The biomimetic NPs formed are highly stable.	
		Membrane hybrids can be formed	
Microfluidic electroporation	The cell membrane suspension and NPs suspension are mixed separately in the instrument, flow through the electroporation area, and finally the product is collected in the chip	High synthesis rate and good parallelism	Complex operation process

**Table 3:** Targeting peptides related to BBB and their protein sequences and corresponding targets.

Target Receptor or Transport Pathway	Name	Peptide Sequence	Ref.
Low-density lipoprotein receptor	Angiopep-2	TFFYGGSRGKRNFKTEEY	[130]
	ApoB	SSVIDALQYKLEGTTRLTRKRGLKLATALSLSNKFVEGS	[131]
	ApoE	LRKLRKRLL	[132]
	mApoE	CWGLRKLRKRLLR	[133]
	Peptide-22	Ac-CMPRLRGC-NH_2_	[134]
Transferrin receptor	B6	CGHKAKGPRK	[135]
	D-T7	d-HRPYIAH	[136]
	T7	HAIYPRH	[136]
	THR	THRPPMWSPVWP-NH_2_	[137]
	THRre	pwvpswmpprht-NH_2_	[138]
	CRT	CRTIGPSVC	[139]
Leptin receptor	Leptin30	YQQILTSMPSRNVIQISNDLENLRDLLHVL	[140]
Nicotinic acetylcholine receptor	RVG29	YTIWMPENPRPGTPCDIFTNSRGKRASNG-OH	[57]
	^D^CDX	GreirtGraerwsekf-OH	[116]
	D8	^D^RTG^D^R^D^A^D^RE^D^W	[141]
Potassium or calcium channel	Apamin	CNCKAPETALCARRCQQH-NH_2_	[142]
	MiniAp-4	H-[Dap]KAPETAL D-NH_2_	[135]
Glutathione transporter	GSH	γ-l-glutamyl-CG-OH	[143]
	G23	HLNILSTLWKYRC	[144]
Adsorption-mediated endocytosis	TAT(47-57)	YGRKKRRQRRR-NH_2_	[145]
	SynB1	RGGRLSYSRRRFSTSTGR	[146]
Unknown receptor	CGN	d-GNHPLAKYNGT	[136]
	TGN	TGNYKALHPHNG	[147]
	TP10	AGYLLGKINLKALAALAKKIL-NH_2_	[148]
Aβ aggregates	LVFFA	LVFFA	[149]
	KLVFF	KLVFF	[64]
	LPFFD	LPFFD	[147]
	QSH	QSHYRHISPAQV	[150]
Sphingomyelin and ganglioside GT1B on neurons	Tet1	HLNILSTLWKYR	[151]

Note: the amino acids are expressed in a single letter; the capital letter represents l-configured amino acids, and the lowercase letters indicate d-type amino acids.

## Data Availability

Not applicable.

## References

[1] Bukhari S.N.A. (2022). Nanotherapeutics for Alzheimer’s disease with preclinical evaluation and clinical trials: Challenges, promises and limitations. Curr. Drug Deliv..

[2] Vermunt L., Sikkes S.A.M., Hout A.V.D., Handels R., Bos I., Flier W.M.V.D., Kern S., Ousset P.J., Maruff P., Skoog I. (2019). Duration of preclinical, prodromal, and dementia stages of Alzheimer’s disease in relation to age, sex, and APOE genotype. Alzheimers Dement..

[3] WHO (2022). Dementia.

[4] Todd S., Barr S., Roberts M., Passmore A.P. (2013). Survival in dementia and predictors of mortality: A review. Int. J. Geriatr. Psychiatry.

[5] Li Y.X., Leng F.D., Xiong Q., Zhou J., Du A.L., Zhu F.Q., Kou X.W., Sun W., Chen L.Z., Wang H.L. (2022). Factors associated with Alzheimer’s disease patients’ caregiving status and family caregiving burden in China. Front. Aging Neurosci..

[6] Gupta G.L., Samant N.P. (2021). Current druggable targets for therapeutic control of Alzheimer’s disease. Contemp. Clin. Trials..

[7] Pardridge W.M. (2001). BBB-genomics: Creating new openings for brain-drug targeting. Drug Discov. Today.

[8] Tsou Y.H., Zhang X.Q., Zhu H., Syed S., Xu X.Y. (2017). Drug delivery to the brain across the blood–brain barrier using nanomaterials. Small.

[9] Tripathi P., Shukla P., Bieberich E. (2022). Theranostic applications of nanomaterials in Alzheimer’s disease: A multifunctional approach. Curr. Pharm. Des..

[10] Chai Z.L., Hu X.F., Lu W.Y. (2017). Cell membrane-coated nanoparticles for tumor-targeted drug delivery. Sci. China Mater..

[11] Jia Y.L., Wang X.B., Hu D.H., Wang P., Liu Q.H., Zhang X.J., Jiang J.Y., Liu X., Sheng Z.H., Liu B. (2018). Phototheranostics: Active targeting of orthotopic glioma using biomimetic proteolipid nanoparticles. ACS Nano.

[12] Wang H.J., Liu Y., He R.Q., Xu D.L., Zang J., Weeranoppanant N., Dong H.Q., Li Y.Y. (2019). Cell membrane biomimetic nanoparticles for inflammation and cancer targeting in drug delivery. Biomater. Sci..

[13] Zou Y., Liu Y.J., Yang Z.P., Zhang D.Y., Lu Y.Q., Zheng M., Xue X., Geng J., Chung R., Shi B.Y. (2018). Effective and targeted human orthotopic glioblastoma xenograft therapy via a multifunctional biomimetic nanomedicine. Adv. Mater..

[14] Zhang W.Y., Zhao M., Gao Y.L., Cheng X., Liu X.Y., Tang S.K., Peng Y.B., Wang N., Hu D.D., Peng H.S. (2021). Biomimetic erythrocytes engineered drug delivery for cancer therapy. Chem. Eng. J..

[15] Cummings J., Lee G., Zhong K., Fonseca J., Taghva K. (2021). Alzheimer’s disease drug development pipeline. Alzheimers Dement..

[16] Melnikova I. (2007). Therapies for Alzheimer’s disease. Nat. Rev. Drug Discov..

[17] Matsunaga S., Kishi T., Nomura I., Sakuma K., Okuya M., Ikuta T., Iwata N. (2018). The efficacy and safety of memantine for the treatment of Alzheimer’s disease. Expert Opin. Drug Saf..

[18] McKeage K. (2009). Memantine: A review of its use in moderate to severe Alzheimer’s disease. CNS Drugs.

[19] Atri A., Hendrix S.B., Pejović V., Hofbauer R.K., Edwards J., Molinuevo J.L., Graham S.M. (2015). Cumulative, additive benefits of memantine-donepezil combination over component monotherapies in moderate to severe Alzheimer’s dementia: A pooled area under the curve analysis. Alzheimer’s Res. Ther..

[20] Dhillon S. (2021). Aducanumab: First approval. Drugs.

[21] Mahase E. (2021). Aducanumab: European agency rejects Alzheimer’s drug over efficacy and safety concerns. The BMJ.

[22] Cano A., Turowski P., Ettcheto M., Duskey J.T., Tosi G., Sánchez-López E., García M.L., Camins A., Souto E.B., Ruiz A. (2021). Nanomedicine-based technologies and novel biomarkers for the diagnosis and treatment of Alzheimer’s disease: From current to future challenges. J. Nanobiotechnology.

[23] Benz F., Liebner S. (2020). Structure and function of the blood-brain barrier (BBB). Handb. Exp. Pharmacol..

[24] Abbott N.J., Rönnbäck L., Hansson E. (2006). Astrocyte–endothelial interactions at the blood–brain barrier. Nat. Rev. Neurosci..

[25] Chowdhury E.A., Noorani B., Alqahtani F., Bhalerao A., Raut S., Sivandzade F., Cucullo L. (2021). Understanding the brain uptake and permeability of small molecules through the BBB: A technical overview. J. Cereb. Blood Flow Metab..

[26] Pardridge W.M. (2005). The blood-brain barrier: Bottleneck in brain drug development. NeuroRx..

[27] Zhao J.J., Ren T.J., Yang M.B., Zhang Y.F., Wang Q.W., Zuo Z. (2019). Reduced systemic exposure and brain uptake of donepezil in rats with scopolamine-induced cognitive impairment. Xenobiotica.

[28] Karasova J.Z., Soukup O., Korabecny J., Hroch M., Krejciova M., Hrabinova M., Misik J., Novotny L., Hepnarova V., Kuca K. (2019). Tacrine and its 7-methoxy derivate; time-change concentration in plasma and brain tissue and basic toxicological profile in rats. Drug Chem. Toxicol..

[29] Ganipineni L.P., Danhier F., Préat V. (2018). Drug delivery challenges and future of chemotherapeutic nanomedicine for glioblastoma treatment. J. Control. Release.

[30] Chatterjee B., Gorain B., Mohananaidu K., Sengupta P., Mandal U.K., Choudhury H. (2019). Targeted drug delivery to the brain via intranasal nanoemulsion: Available proof of concept and existing challenges. Int. J. Pharm..

[31] Lu X., Zhang Y., Wang L., Li G., Gao J., Wang Y. (2021). Development of L-carnosine functionalized iron oxide nanoparticles loaded with dexamethasone for simultaneous therapeutic potential of blood brain barrier crossing and ischemic stroke treatment. Drug Deliv..

[32] Pinheiro R.G.R., Granja A., Loureiro J.A., Pereira M.C., Pinheiro M., Neves A.R., Reis S. (2020). RVG29-functionalized lipid nanoparticles for quercetin brain delivery and Alzheimer’s disease. Pharm. Res..

[33] Yusuf M., Khan M., Alrobaian M.M., Alghamdi S.A., Warsi M.H., Sultana S., Khan R.A. (2020). Brain targeted polysorbate-80 coated PLGA thymoquinone nanoparticles for the treatment of Alzheimer’s disease, with biomechanistic insights. J. Drug Deliv. Sci. T echnol..

[34] Meng Q., Wang A., Hua H., Jiang Y., Wang Y., Mu H., Wu Z., Sun K. (2018). Intranasal delivery of huperzine A to the brain using lactoferrin-conjugated N-trimethylated chitosan surface-modified PLGA nanoparticles for treatment of Alzheimer’s disease. Int. J. Nanomed.

[35] Xu R., Wang J., Xu J., Song X., Huang H., Feng Y., Fu C. (2020). Rhynchophylline loaded-mPEG-PLGA nanoparticles coated with Tween-80 for preliminary study in Alzheimer’s disease. Int. J. Nanomed..

[36] Barbalace M.C., Malaguti M., Giusti L., Lucacchini A., Hrelia S., Angeloni C. (2019). Anti-inflammatory activities of marine algae in neurodegenerative diseases. Int. J. Mol. Sci..

[37] Wang C., Du W., Lu H., Lan J., Liang K., Cao S. (2021). A review: Halogenated compounds from marine actinomycetes. Molecules.

[38] Vio V., Marchant M.J., Araya E., Kogan M.J. (2017). Metal nanoparticles for the treatment and diagnosis of neurodegenerative brain diseases. Curr. Pharm. Des..

[39] Bayda S., Adeel M., Tuccinardi T., Cordani M., Rizzolio F. (2019). The history of nanoscience and nanotechnology: From chemical–physical applications to nanomedicine. Molecules.

[40] Joy R., George J., John F. (2022). Brief outlook on polymeric nanoparticles, micelles, niosomes, hydrogels and liposomes: Preparative methods and action. ChemistrySelect.

[41] Tosi G., Duskey J.T., Kreuter J. (2019). Nanoparticles as carriers for drug delivery of macromolecules across the blood-brain barrier. Expert Opin. Drug. Deliv..

[42] Danhier F., Ansorena E., Silva J.M., Coco R., Breton A.L., Préat V. (2012). PLGA-based nanoparticles: An overview of biomedical applications. J. Control. Release.

[43] Khan A.R., Yang X.Y., Fu M.F., Zhai G.X. (2018). Recent progress of drug nanoformulations targeting to brain. J. Control. Release.

[44] Rocha C.V., Gonçalves V., Silva M.C.D., Bañobre-López M., Gallo J. (2022). PLGA-based composites for various biomedical applications. Int. J. Mol. Sci..

[45] Khan I., Gothwal A., Sharma A.K., Kesharwani P., Gupta L., Lyer A.K., Gupta U. (2016). PLGA nanoparticles and their versatile role in anticancer drug delivery. Crit. Rev. Ther. Drug Carrier Syst..

[46] Gao C., Chu X., Gong W., Zheng J., Xie X., Wang Y., Yang M., Li Z., Gao C., Yang Y. (2020). Neuron tau-targeting biomimetic nanoparticles for curcumin delivery to delay progression of Alzheimer’s disease. J. Nanobiotechnology.

[47] Tang D., Wang Y., Wijaya A., Liu B.Y., Maruf A., Wang J.X., Xu J.X., Liao X.L., Wu W., Wang G.X. (2021). ROS-responsive biomimetic nanoparticles for potential application in targeted anti-atherosclerosis. Regen Biomater..

[48] Perche F., Uchida S., Akiba H., Lin C.Y., Ikegami M., Dirisala A., Nakashima T., Itaka K., Tsumoto K., Kataoka K. (2016). Improved brain expression of anti-amyloid β scFv by complexation of mRNA including a secretion sequence with PEG-based block catiomer. Curr. Alzheimer Res..

[49] Xie J., Gonzalez-Carter D., Tockary T.A., Nakamura N., Xue Y., Nakakido M., Akiba H., Dirisala A., Liu X., Toh K. (2020). Dual-sensitive nanomicelles enhancing systemic delivery of therapeutically active antibodies specifically into the brain. ACS Nano.

[50] Gonzalez-Carter D., Liu X., Tockary T.A., Dirisala A., Toh K., Anraku Y., Kataoka K. (2020). Targeting nanoparticles to the brain by exploiting the blood-brain barrier impermeability to selectively label the brain endothelium. Proc. Natl. Acad. Sci. USA.

[51] Akbarzadeh A., Rezaei-Sadabady R., Davaran S., Joo S.W., Zarghami N., Hanifehpour Y., Samiei M., Kouhi M., Nejati-Koshki K. (2013). Liposome: Classification, preparation, and applications. Nanoscale Res. Lett..

[52] Tulbah A.S., Lee W.H. (2021). Physicochemical characteristics and in vitro toxicity/anti-SARS-CoV-2 activity of favipiravir solid lipid nanoparticles (SLNs). Pharmaceuticals.

[53] Khosa A., Reddi S., Saha R.N. (2018). Nanostructured lipid carriers for site-specific drug delivery. Biomed. Pharmacother..

[54] Wilson R.J., Li Y., Yang G.Z., Zhao C.X. (2021). Nanoemulsions for drug delivery. Particuology.

[55] Daraee H., Etemadi A., Kouhi M., Alimirzalu S., Akbarzadeh A. (2014). Application of liposomes in medicine and drug delivery. Artif. Cells Nanomed. Biotechnol..

[56] Song Y.N., Zhang N., Li Q.Y., Chen J., Wang Q.Z., Yang H.B., Tan H.P., Gao J.F., Dong Z.H., Pang Z.Q. (2020). Biomimetic liposomes hybrid with platelet membranes for targeted therapy of atherosclerosis. Chem. Eng. J..

[57] Han Y., Gao C.H., Wang H., Sun J.J., Liang M., Feng Y., Liu Q.Q., Fu S.Y., Cui L., Gao C.S. (2021). Macrophage membrane-coated nanocarriers Co-modified by RVG29 and TPP improve brain neuronal mitochondria-targeting and therapeutic efficacy in Alzheimer’s disease mice. Bioact. Mater..

[58] Gao N., Sun H.J., Dong K., Ren J.S., Qu X.G. (2014). Gold-nanoparticle-based multifunctional amyloid-β inhibitor against Alzheimer’s disease. Chem. Eur. J..

[59] Tengjisi, Hui Y., Yang G.Z., Fu C.K., Liu Y., Zhao C.X. (2020). Biomimetic core-shell silica nanoparticles using a dual-functional peptide. J. Colloid Interface Sci..

[60] Nakamura S., Sato M., Sato Y., Ando N., Takayama T., Fujita M., Ishihara M. (2019). Synthesis and application of silver nanoparticles (Ag NPs) for the prevention of infection in healthcare workers. Int. J. Mol. Sci..

[61] Liu D.Z., Cheng Y., Cai R.Q., Wang W.W., Cui H., Liu M., Zhang B.L., Mei Q.B., Zhou S.Y. (2018). The enhancement of siPLK1 penetration across BBB and its anti glioblastoma activity in vivo by magnet and transferrin co-modified nanoparticle. Nanomedicine.

[62] Ehlerding E.B., Chen F., Cai W.B. (2015). Biodegradable and renal clearable inorganic nanoparticles. Adv. Sci..

[63] Yang G.B., Phua S.Z.F., Lim W.Q., Zhang R., Feng L.Z., Liu G.F., Wu H.W., Bindra A.K., Jana D., Liu Z. (2019). A hypoxia-responsive albumin-based nanosystem for deep tumor penetration and excellent therapeutic efficacy. Adv. Mater..

[64] Plissonneau M., Pansieri J., Heinrich-Balard L., Morfin J.F., Stransky-Heilkron N., Rivory P., Mowat P., Dumoulin M., Cohen R., Allémann É. (2016). Gd-nanoparticles functionalization with specific peptides for ß-amyloid plaques targeting. J. Nanobiotechnology.

[65] Pooja D., Tunki L., Kulhari H., Reddy B.B., Sistla R. (2016). Optimization of solid lipid nanoparticles prepared by a single emulsification-solvent evaporation method. Data Brief.

[66] Cortesi R., Esposjto E., Luca G., Nastruzzi C. (2002). Production of lipospheres as carriers for bioactive compounds. Biomaterials.

[67] Si W., Yang Q., Zong Y., Ren G., Zhao L., Hong M., Xin Z. (2021). Toward understanding the effect of solvent evaporation on the morphology of PLGA microspheres by double emulsion method. Ind. Eng. Chem. Res..

[68] Almoustafa H.A., Alshawsh M.A., Chik Z. (2017). Technical aspects of preparing PEG-PLGA nanoparticles as carrier for chemotherapeutic agents by nanoprecipitation method. Int. J. Pharm..

[69] Sheth P., Sandhu H., Singhal D., Malick W., Shah N., Kislalioglu M.S. (2012). Nanoparticles in the pharmaceutical industry and the use of supercritical fluid technologies for nanoparticle production. Curr. Drug Deliv..

[70] Maqbool F., Moyle P.M., Thurecht K.J., Falconer J.R. (2019). Dispersibility of phospholipids and their optimization for the efficient production of liposomes using supercritical fluid technology. Int. J. Pharm..

[71] Islam T., Ragib A.A., Ferdosh S., Uddin A.B.M.H., Akanda M.J.H., Mia M.A.R., M R.P.D., Kamaruzzaman B.Y., Sarker M.Z.I. (2022). Development of nanoparticles for pharmaceutical preparations using supercritical techniques. Chem. Eng. Commun..

[72] Ali M.E., Lamprecht A. (2016). Spray freeze drying as an alternative technique for lyophilization of polymeric and lipid-based nanoparticles. Int. J. Pharm..

[73] Lee S.H., Heng D., Ng W.K., Chan H.-K., Tan R.B.H. (2011). Nano spray drying: A novel method for preparing protein nanoparticles for protein therapy. Int. J. Pharm..

[74] Li R.X. (2020). Progress in preparation methods and applications of inorganic nanoparticles. J. Phys. Conf. Ser..

[75] Li Y., Han Q.L., Yao Y., Li M., Dong P., Han L., Zeng X.Y., Liu J., Liu J.M., Zhang Y.J. (2019). Comparative study of yttria-stabilized zirconia synthesis by Co-precipitation and solvothermal methods. JOM.

[76] Mousavi S.H., Müller T.S., Karos R., Oliveira P.W.D. (2016). Faster synthesis of CIGS nanoparticles using a modified solvothermal method. J. Alloys Compd..

[77] Chun Y.G., Kim K.H., Yoon K.H. (2005). Synthesis of CuLnGaSe2 nanoparticles by solvothermal route. Thin Solid Films.

[78] Lismont M., Páez C.A., Dreesen L. (2015). A one-step short-time synthesis of [email protected]2 core–shell nanoparticles. J. Colloid Interface Sci..

[79] Sheng S., Jin S., Cui K. (2020). Thermal decomposition of nanostructured bismuth subcarbonate. Materials.

[80] Oroojalian F., Beygi M., Baradaran B., Mokhtarzadeh A., Shahbazi M.A. (2021). Immune cell membrane-coated biomimetic nanoparticles for targeted cancer therapy. Small.

[81] Chen L., Hong W.Q., Ren W.Y., Xu T., Qian Z.Y., He Z.Y. (2021). Recent progress in targeted delivery vectors based on biomimetic nanoparticles. Signal Transduct. Target. Ther..

[82] Huang Y.X., Tuo W.W., Wang D., Kang L.L., Chen X.Y., Luo M. (2015). Restoring the youth of aged red blood cells and extending their lifespan in circulation by remodelling membrane sialic acid. J. Cell. Mol. Med..

[83] Dehaini D., Wei X.L., Fang R.H., Masson S., Angsantikul P., Luk B.T., Zhang Y., Ying M., Jiang Y., Kroll A.V. (2017). Erythrocyte–platelet hybrid membrane coating for enhanced nanoparticle functionalization. Adv. Mater..

[84] Zhao H., Song S.S., Ma J.W., Yan Z.Y., Xie H.W., Feng Y., Che S.S. (2022). CD47 as a promising therapeutic target in oncology. Front. Immunol..

[85] Hu C.M.J., Fang R.H., Zhang L. (2012). Erythrocyte-inspired delivery systems. Adv. Healthc. Mater..

[86] Liu B., Wang W.M., Fan J.L., Long Y., Xiao F., Daniyal M., Tong C.Y., Xie Q., Jian Y.Q., Li B. (2019). RBC membrane camouflaged prussian blue nanoparticles for gamabutolin loading and combined chemo/photothermal therapy of breast cancer. Biomaterials.

[87] Gao C.H., Wang Y.L., Sun J.J., Han Y., Gong W., Li Y., Feng Y., Wang H., Yang M.Y., Li Z.P. (2020). Neuronal mitochondria-targeted delivery of curcumin by biomimetic engineered nanosystems in Alzheimer’s disease mice. Acta Biomater..

[88] Meijden P.E.J.V.D., Heemskerk J.W.M. (2019). Platelet biology and functions: New concepts and clinical perspectives. Nat. Rev. Cardiol..

[89] Avecilla S.T., Hattori K., Heissig B., Tejada R., Liao F., Shido K., Jin D.K., Dias S., Zhang F., Hartman T.E. (2004). Chemokine-mediated interaction of hematopoietic progenitors with the bone marrow vascular niche is required for thrombopoiesis. Nat. Med..

[90] Koupenova M., Clancy L., Corkrey H.A., Freedman J.E. (2018). Circulating platelets as mediators of immunity, inflammation, and thrombosis. Circ. Res..

[91] Rayes J., Watson S.P., Nieswandt B. (2019). Functional significance of the platelet immune receptors GPVI and CLEC-2. J. Clin. Investig..

[92] McDonald B., Dunbar M. (2019). Platelets and intravascular immunity: Guardians of the vascular space during bloodstream infections and sepsis. Front. Immunol..

[93] Xu J.P., Wang X.Q., Yin H.Y., Cao X., Hu Q.Y., Lv W., Xu Q.W., Gu Z., Xin H.L. (2019). Sequentially site-specific delivery of thrombolytics and neuroprotectant for enhanced treatment of ischemic stroke. ACS Nano.

[94] Zinger A., Soriano S., Baudo G., Rosa E.D., Taraballi F., Villapol S. (2021). Biomimetic nanoparticles as a theranostic tool for traumatic brain injury. Adv. Funct. Mater..

[95] Martinez J.O., Molinaro R., Hartman K.A., Boada C., Sukhovershin R., Rosa E.D., Kuri D., Zhang S.R., Evangelopoulos M., Carter A.M. (2018). Biomimetic nanoparticles with enhanced affinity towards activated endothelium as versatile tools for theranostic drug delivery. Theranostics.

[96] Li R., He Y., Zhang S., Qin J., Wang J. (2017). Cell membrane-based nanoparticles: A new biomimetic platform for tumor diagnosis and treatment. Acta Pharm. Sin. B.

[97] Feng L.S., Dou C.R., Xia Y.G., Li B.H., Zhao M.Y., Yu P., Zheng Y.Y., El-Toni A.M., Atta N.F., Galal A. (2021). Neutrophil-like cell-membrane-coated nanozyme therapy for ischemic brain damage and long-term neurological functional recovery. ACS Nano.

[98] Liu R., An Y., Jia W.F., Wang Y.S., Wu Y., Zhen Y.H., Cao J., Gao H.L. (2020). Macrophage-mimic shape changeable nanomedicine retained in tumor for multimodal therapy of breast cancer. J. Control. Release.

[99] Pang L., Qin J., Han L., Zhao W., Liang J., Xie Z., Yang P., Wang J. (2016). Exploiting macrophages as targeted carrier to guide nanoparticles into glioma. Oncotarget.

[100] Yurdagul A., Finney A.C., Woolard M.D., Orr A.W. (2016). The arterial microenvironment: The where and why of atherosclerosis. Biochem. J..

[101] Nagenborg J., Goossens P., Biessen E.A.L., Donners M.M.P.C. (2017). Heterogeneity of atherosclerotic plaque macrophage origin, phenotype and functions: Implications for treatment. Eur. J. Pharmacol..

[102] Long Y., Xiang Y., Liu S.Y., Zhang Y.L., Wan J.Y., Ci Z.M., Cui M.Q., Shen L., Li N., Guan Y.M. (2022). Macrophage membrane modified baicalin liposomes improve brain targeting for alleviating cerebral ischemia reperfusion injury. Nanomedicine.

[103] Sun H., Su J., Meng Q., Yin Q., Chen L., Gu W., Zhang P., Zhang Z., Yu H., Wang S. (2016). Cancer-cell-biomimetic nanoparticles for targeted therapy of homotypic tumors. Adv. Mater..

[104] Li J., Wang X.D., Zheng D.Y., Lin X.Y., Wei Z.W., Zhang D., Li Z.F., Zhang Y., Wu M., Liu X.L. (2018). Cancer cell membrane-coated magnetic nanoparticles for MR/NIR fluorescence dual-modal imaging and photodynamic therapy†. Biomater. Sci..

[105] Drolez A., Vandenhaute E., Julien S., Gosselet F., Burchell J., Cecchelli R., Delannoy P., Dehouck M.-P., Mysiorek C. (2016). Selection of a relevant in vitro blood-brain barrier model to investigate pro-metastatic features of human breast cancer cell lines. PLoS ONE.

[106] Wang C.X., Wu B., Wu Y.T., Song X.Y., Zhang S.S., Liu Z.H. (2020). Camouflaging nanoparticles with brain metastatic tumor cell membranes: A new strategy to traverse blood–brain barrier for imaging and therapy of brain tumors. Adv. Funct. Mater..

[107] Zhao Y.N., Li A., Jiang L.D., Gu Y.W., Liu J.Y. (2021). Hybrid membrane-coated biomimetic nanoparticles (HM@BNPs): A multifunctional nanomaterial for biomedical applications. Biomacromolecules.

[108] Ai X.Z., Wang S.Y., Duan Y.O., Zhang Q.Z., Chen M.S., Gao W.W., Zhang L.F. (2020). Emerging approaches to functionalizing cell membrane-coated nanoparticles. Biochemistry.

[109] Yin Y., Tang W., Ma X.Y., Tang L., Zhang Y., Yang M., Hu F.F., Li G.L., Wang Y.Z. (2021). Biomimetic neutrophil and macrophage dual membrane-coated nanoplatform with orchestrated tumor-microenvironment responsive capability promotes therapeutic efficacy against glioma. Chem. Eng. J..

[110] Niu W.B., Xiao Q., Wang X.J., Zhu J.Q., Li J.H., Liang X.M., Peng Y.M., Wu C.T., Lu R.J., Pan Y. (2021). A biomimetic drug delivery system by integrating grapefruit extracellular vesicles and doxorubicin-loaded heparin-based nanoparticles for glioma therapy. Nano Lett..

[111] Hao Q., Liu Q.H., Wang X.B., Wang P., Li T., Tong W.Y. (2009). Membrane damage effect of therapeutic ultrasound on Ehrlich ascitic tumor cells. Cancer Biother. Radiopharm..

[112] Fabiilli M.L., Haworth K.J., Fakhri N.H., Kripfgans O.D., Carson P.L., Fowlkes J.B. (2009). The role of inertial cavitation in acoustic droplet vaporization. IEEE Trans. Ultrason. Ferroelectr. Freq. Control.

[113] Wang W.J., Chen W.J., Zou M.M., Lv R.L., Wang D.L., Hou F.R., Feng H., Ma X.B., Zhong J.J., Ding T. (2018). Applications of power ultrasound in oriented modification and degradation of pectin: A review. J. Food Eng..

[114] Amini M., Niemi E., Hisdal J., Kalvøy H., Tronstad C., Scholz H., Rosales A., Martinsen Ø.G. (2020). Monitoring the quality of frozen-thawed venous segments using bioimpedance spectroscopy. Physiol. Meas..

[115] Fu Q., Lv P.P., Chen Z.K., Ni D.Z., Zhang L.J., Yue H., Yue Z.G., Wei W., Ma G.H. (2015). Programmed co-delivery of paclitaxel and doxorubicin boosted by camouflaging with erythrocyte membrane†. Nanoscale.

[116] Chai Z.L., Hu X.F., Wei X.L., Zhan C.Y., Lu L.W., Jiang K., Su B.X., Ruan H.T., Ran D.N., Fang R.H. (2017). A facile approach to functionalizing cell membrane-coated nanoparticles with neurotoxin-derived peptide for brain-targeted drug delivery. J. Control. Release.

[117] Klenk C., Heim D., Ugele M., Hayden O. (2019). Impact of sample preparation on holographic imaging of leukocytes. Opt. Eng..

[118] Kang T., Zhu Q.Q., Wei D., Feng J.X., Yao J.H., Jiang T.Z., Song Q.X., Wei X.B., Chen H.Z., Gao X.L. (2017). Nanoparticles coated with neutrophil membranes can effectively treat cancer metastasis. ACS Nano.

[119] Barua P., Ahn S.B., Mohamedali A., Liu F. (2020). Improved sensitivity in cell surface protein detection by combining chemical labeling with mechanical lysis in a colorectal cancer cell model. Biotechnol. Lett..

[120] DeCaprio J., Kohl T.O. (2019). Using Dounce homogenization to lyse cells for immunoprecipitation. Cold Spring Harb. Protoc..

[121] Rahman M.A., Wang J., Zhang C., Olah A., Baer E. (2016). Novel micro-/nano- porous cellular membranes by forced assembly co-extrusion technology. Eur. Polym. J..

[122] He W.P., Frueh J., Wu Z.W., He Q. (2016). Leucocyte membrane-coated janus microcapsules for enhanced photothermal cancer treatment. Langmuir.

[123] Movahed S., Li D.Q. (2010). Microfluidics cell electroporation. Microfluid Nanofluidics.

[124] Rao L., Cai B., Bu L.L., Liao Q.Q., Guo S.S., Zhao X.Z., Dong W.F., Liu W. (2017). Microfluidic electroporation-facilitated synthesis of erythrocyte membrane-coated magnetic nanoparticles for enhanced imaging-guided cancer therapy. ACS Nano.

[125] Huang D., Zhao D.Y., Li J.H., Wu Y.T., Zhou W.B., Wang W., Liang Z.C., Li Z.H. (2017). High cell viability microfluidic electroporation in a curved channel. Sens. Actuators B Chem..

[126] Marqués-Gallego P., Kroon A.I.P.M.d. (2014). Ligation strategies for targeting liposomal nanocarriers. BioMed. Res. Int..

[127] García-Granados R., Lerma-Escalera J.A., Morones-Ramírez J.R. (2019). Metabolic engineering and synthetic biology: Synergies, future, and challenges. Front. Bioeng. Biotechnol..

[128] Stephan M.T., Irvine D.J. (2011). Enhancing cell therapies from the outside in: Cell surface engineering using synthetic nanomaterials. Nano Today.

[129] Zhou H., Fan Z.Y., Lemons P.K., Cheng H. (2016). A facile approach to functionalize cell membrane-coated nanoparticles. Theranostics.

[130] Wang R.R., Wang C.W., Dai Z.Z., Chen Y.Z., Shen Z.W., Xiao G., Chen Y.F., Zhou J.N., Zhuang Z.R., Wu R.H. (2019). An amyloid-β targeting chemical exchange saturation transfer probe for in vivo detection of Alzheimer’s disease. ACS Chem. Neurosci..

[131] Spencer B., Trinh L., Rockenstein E., Mante M., Florio J., Adame A., El-Agnaf O.M.A., Kim C., Masliah E., Rissman R.A. (2019). Systemic peptide mediated delivery of an siRNA targeting α-syn in the CNS ameliorates the neurodegenerative process in a transgenic model of Lewy body disease. Neurobiol. Dis..

[132] Topal G.R., Mészáros M., Porkoláb G., Szecskó A., Polgár T.F., Siklós L., Deli M.A., Veszelka S., Bozkir A. (2020). ApoE-targeting increases the transfer of solid lipid nanoparticles with donepezil cargo across a culture model of the blood–brain barrier. Pharmaceutics.

[133] Corti R., Cox A., Cassina V., Nardo L., Salerno D., Marrano C.A., Missana N., Andreozzi P., Silva P.J., Stellacci F. (2020). The clustering of mApoE anti-amyloidogenic peptide on nanoparticle surface does not alter its performance in controlling beta-amyloid aggregation. Int. J. Mol. Sci..

[134] Séguy L., Guyon L., Maurel M., Verdié P., Davis A., Corvaisier S., Lisowski V., Dallemagne P., Groo A.C., Malzert-Fréon A. (2021). Active targeted nanoemulsions for repurposing of tegaserod in Alzheimer’s disease treatment. Pharmaceutics.

[135] Israel L.L., Galstyan A., Cox A., Shatalova E.S., Sun T., Rashid M.-H., Grodzinski Z., Chiechi A., Fuchs D.-T., Patil R. (2022). Signature effects of vector-guided systemic nano bioconjugate delivery across blood-brain barrier of normal, Alzheimer’s, and tumor mouse models. ACS Nano.

[136] Hou Q.H., Zhu L.N., Wang L., Liu X.Y., Xiao F., Xie Y.Z.X., Zheng W.F., Jiang X.Y. (2022). Screening on-chip fabricated nanoparticles for penetrating the blood–brain barrier. Nanoscale.

[137] Zhang X.T., He T., Chai Z., Samulski R.J., Li C.W. (2018). Blood-brain barrier shuttle peptides enhance AAV transduction in the brain after systemic administration. Biomaterials.

[138] Díaz-Perlas C., Oller-Salvia B., Sánchez-Navarro M., Teixidó M., Giralt E. (2018). Branched BBB-shuttle peptides: Chemoselective modification of proteins to enhance blood–brain barrier transport†. Chem. Sci..

[139] Falanga A., Melone P., Cagliani R., Borbone N., D’Errico S., Piccialli G., Netti P., Guarnieri D. (2018). Design, synthesis and characterization of novel co-polymers decorated with peptides for the selective nanoparticle transport across the cerebral endothelium. Molecules.

[140] Liu Y., Li J.F., Shao K., Huang R.Q., Ye L.Y., Lou J.N., Jiang C. (2010). A leptin derived 30-amino-acid peptide modified pegylated poly-L-lysine dendrigraft for brain targeted gene delivery. Biomaterials.

[141] Farshbaf M., Mojarad-Jabali S., Hemmati S., Khosroushahi A.Y., Motasadizadeh H., Zarebkohan A., Valizadeh H. (2022). Enhanced BBB and BBTB penetration and improved anti-glioma behavior of bortezomib through dual-targeting nanostructured lipid carriers. J. Control. Release.

[142] Fattahi H., Esmaeil N., Aliomrani M. (2021). Apamin as a BBB shuttle and its effects on T cell population during the experimental autoimmune encephalomyelitis-induced model of multiple sclerosis. Neurotox. Res..

[143] Najmi A., Wang S.G., Huang Y., Seefeldt T., Alqahtani Y., Guan X.M. (2021). 2-(2-Cholesteroxyethoxyl)ethyl 3′-S-glutathionylpropionate and its self-assembled micelles for brain delivery: Design, synthesis and evaluation. Int. J. Pharm..

[144] Zhang H.Y., Os W.L.v., Tian X.B., Zu G.Y., Ribovski L., Bron R., Bussmann J., Kros A., Liu Y., Zuhorn I.S. (2021). Development of curcumin-loaded zein nanoparticles for transport across the blood–brain barrier and inhibition of glioblastoma cell growth. Biomater. Sci..

[145] Maderna E., Colombo L., Cagnotto A., Fede G.D., Indaco A., Tagliavini F., Salmona M., Giaccone G. (2018). In situ tissue labeling of cerebral amyloid using HIV-related Tat peptide. Mol. Neurobiol..

[146] Rousselle C., Clair P., Temsamani J., Scherrmann J.-M. (2002). Improved brain delivery of benzylpenicillin with a peptide-vector-mediated strategy. J. Drug Target..

[147] Yang L.C., Sun J., Xie W.J., Liu Y.N., Liu J. (2017). Dual-functional selenium nanoparticles bind to and inhibit amyloid β fiber formation in Alzheimer’s disease. J. Mater. Chem. B.

[148] Rusiecka I., Ruczyński J., Kozłowska A., Backtrog E., Mucha P., Kocić I., Rekowski P. (2019). TP10-dopamine conjugate as a potential therapeutic agent in the treatment of Parkinson’s disease. Bioconjug. Chem..

[149] Loureiro J.A., Gomes B., Fricker G., Coelho M.A.N., Rocha S., Pereira M.C. (2016). Cellular uptake of PLGA nanoparticles targeted with anti-amyloid and anti-transferrin receptor antibodies for Alzheimer’s disease treatment. Colloids Surf. B.

[150] Zhang C., Wan X., Zheng X.Y., Shao X.Y., Liu Q.F., Zhang Q.Z., Qian Y. (2014). Dual-functional nanoparticles targeting amyloid plaques in the brains of Alzheimer’s disease mice. Biomaterials.

[151] Guo Q., Xu S.T., Yang P., Wang P.Z., Lu S., Sheng D.Y., Qian K., Cao J.X., Lu W., Zhang Q.Z. (2020). A dual-ligand fusion peptide improves the brain-neuron targeting of nanocarriers in Alzheimer’s disease mice. J. Control. Release.

